# Advancing Pharm. D. Training in Egypt through a Structured Preceptor Development Program

**DOI:** 10.3390/pharmacy8030135

**Published:** 2020-08-01

**Authors:** Toral C. Patel, Jodie V. Malhotra, Joseph J. Saseen

**Affiliations:** Department of Clinical Pharmacy, Skaggs School of Pharmacy and Pharmaceutical Sciences, University of Colorado Anschutz Medical Campus, Aurora, CO 80045, USA; jodie.malhotra@cuanschutz.edu (J.V.M.); joseph.saseen@cuanschutz.edu (J.J.S.)

**Keywords:** continuing professional development, continuing education, pharmacy education, pharmacy workforce development, pharmacy practice change management

## Abstract

The Children’s Cancer Hospital of Egypt (CCHE) and the University of Colorado Skaggs School of Pharmacy and Pharmaceutical Sciences (SSPPS) collaborate to offer a Doctor of Pharmacy (Pharm.D.) degree to international pharmacists holding a bachelor’s degree in pharmacy. The experiential training is provided by CCHE’s clinical pharmacist preceptors at CCHE. Clinical pharmacists at CCHE had prior experience precepting baccalaureate pharmacy students, but not Pharm.D. students when this program commenced. Therefore, the SSPPS faculty provided a live preceptor development program for select CCHE clinical pharmacists in 2017. Primary deliverables of the program included the preparation of individual preceptor development plans and experiential syllabi for program participants. Preceptor development plans and experiential syllabi were evaluated by the SSPPS faculty. Program participants were also evaluated on their assessment of learner case scenarios using introductory pharmacy practice experience (IPPE) and advanced pharmacy practice experience (APPE) assessment tools created for the CCHE program. Participant performance on submitted preceptor development plans and experiential syllabi, and performance on the learner cases were all utilized for participant selection as Pharm.D. preceptors in the CCHE Pharm.D. program. This paper describes this preceptor development program, the process utilized to determine selection of Pharm.D. preceptors, and plans for providing continuing preceptor development for preceptors at CCHE.

## 1. Introduction

As the global need for advanced clinical pharmacy services is recognized, an increasing number of international programs have developed post-baccalaureate Doctor of Pharmacy (Pharm.D.) degree (or similar) programs to meet those needs. However, the didactic curricula and experiential training within these international post-graduate Pharm.D. programs vary significantly and many lack qualified and experienced clinical pharmacists and practice sites to facilitate proper experiential training for Pharm.D. students to provide advanced clinical pharmacy services [1,2,3,4,5]. Doctor of Pharmacy students need opportunities to practice clinical pharmacy with mentorship and supervision provided by qualified clinical pharmacist preceptors to ensure the appropriate development and provision of clinical pharmacy services independently. Experiential sites need a process to identify the best and most capable preceptors among their existing pharmacists to train Pharm.D. students and a process to provide continuing professional development of preceptor skills for their preceptors. Standards do not currently exist on how to develop and select pharmacists to serve as Pharm.D. preceptors [6]. This paper describes the preceptor development training program, preceptor selection methods, and continuing preceptor development for Pharm.D. preceptors at the Children’s Cancer Hospital Egypt (CCHE).

In 2016, CCHE, a comprehensive pediatric cancer hospital and leader of clinical innovation in the region, and the University of Colorado Skaggs School of Pharmacy and Pharmaceutical Sciences (SSPPS), nationally recognized for educational innovation and producing excellent pharmacists for over 100 years, entered into a memorandum of understanding. The two institutions partnered to create a Pharm.D. program offered by CCHE in collaboration with the University of Colorado Skaggs School of Pharmacy and Pharmaceutical Sciences (SSPPS) to provide baccalaureate-trained pharmacists with state-of-the-art didactic and clinical education. The SSPPS faculty with experience in Pharm.D. program design and implementation guided the CCHE in the development of the mission, vision, outcomes, curriculum, policies, procedures, and administrative services for the Pharm.D. program. This program started in 2017. The experiential training for the CCHE/SSPPS Pharm.D. program takes place at the CCHE in Egypt.

Other pharmacy programs within the United States have described international collaborations expanding Pharm.D. training internationally. The University of Purdue, College of Pharmacy supports a clinical practice site in Kenya for advanced pharmacy practice experience (APPE) rotations for students from Purdue and Kenyan pharmacy interns since 2004 [7,8]. The University of Malta and the University of Illinois at Chicago describe a similar collaboration to the CCHE/SSPPS PharmD. program where the experiential training takes place primarily in Malta [9,10]. The CCHE/SSPPS program is unique in that development of pharmacists in the partnering organization has been a primary objective within the collaboration.

## 2. CCHE/SSPPS Collaborative Pharm.D. Program

An inaugural class of 8 students enrolled into the CCHE/SSPPS Pharm.D. program in May 2018. The program is a two-year post-baccalaureate PharmD program consisting of six sequential semesters. The didactic portion of the curriculum is offered online by the SSPPS faculty while the experiential training is completed at the CCHE in Cairo, Egypt. The objectives for the experiential rotations for the CCHE/SSPPS Pharm.D. program are modeled on introductory pharmacy practice experiences (IPPE) and advanced pharmacy practice experiences (APPE), similar to those required in Pharm.D. programs in the United States. The experiential curriculum consists of IPPE rotations to be completed over the first four semesters (90 h per semester) followed by six APPE rotations (total 1200 h) to be completed over the following two semesters. Each APPE rotation length is five weeks. Each student is required to complete two assigned APPE rotations in inpatient general medicine, one assigned APPE rotation in ambulatory care, and three elective APPE rotations.

The CCHE is an ideal experiential site for Pharm.D. students to gain exposure to multiple advanced clinical pharmacy services provided by experienced clinical pharmacists. In the United States, the Accreditation Council for Pharmacy Education (ACPE) accreditation standards for experiential training [11] emphasize interprofessional collaborations in pharmacy practice utilizing the Joint Commission of Pharmacy Practitioners’ Patient Care Process (PPCP) [12]. The CCHE provides advanced clinical pharmacy services to pediatric oncology patients in inpatient, ambulatory, research, and transitional settings. Clinical pharmacists at the CCHE work directly with physicians, nurses, and patients to develop and implement optimal pharmacotherapy care plans for patients in inpatient, ambulatory, and transitional settings. The interprofessional model of care at the CCHE aligns with the Joint Commission of Pharmacy Practitioners’ Patient Care Process (PPCP) [12]. The clinical pharmacists at CCHE are experts within their practice areas and are experienced preceptors. Many clinical pharmacists at the CCHE hold Board of Pharmacy Specialties Board Certifications within their specialty practice area(s).

Preceptors at the CCHE were already experienced at precepting baccalaureate degree pharmacy students prior to the start of the Pharm.D. program at the CCHE. Preparation to precept Pharm.D. students for IPPEs and APPEs required for the CCHE/SSPPS post-graduate Pharm.D. program, was a new expectation and requirement within the CCHE. The CCHE identified many potential Pharm.D. preceptors within their current pharmacist preceptors; however, none of the identified pharmacists held a Pharm.D. degree or had prior experience precepting Pharm.D. students. Therefore, the CCHE and SSPPS felt these pharmacists would benefit from formal preceptor development to prepare them to precept Pharm.D. students.

## 3. Materials and Methods

In 2017, three SSPPS faculty experienced in the development and precepting of Pharm.D. students facilitated a 5-day live preceptor development program at CCHE. Participants were selected from the CCHE clinical pharmacists group based on their potential to be Pharm.D. preceptors by the CCHE based on prior success precepting baccalaureate degree pharmacy students. The objectives developed for the preceptor training program included: assess preceptor practice sites for opportunities for student engagement, design learning outcomes and a plan for experiential rotations, examine learning styles and techniques for precepting, differentiate formative and summative assessment processes, implement effective techniques to provide constructive feedback, and identify challenging student situations and resolution strategies.

The schedule for the preceptor development program is summarized in Table 1. Content was, identified to enhance preceptor capabilities, and provide a broad overview of contemporary clinical pharmacy practice skills and abilities. Lectures, active learning, video case-discussions, and reflection activities were utilized to facilitate learning.

Program activities and assignments enabled participants to create a personal continuing preceptor development plan and a rotation syllabus for their proposed Pharm.D. rotation. Creation of an individual preceptor development plan was incorporated as a key outcome for the program to help participants match strategies covered within the program with self-identified areas of interest for preceptor development as they embark on their new roles as Pharm.D. preceptors and to contribute to participant continuing professional development (CPD). CPD, is a cyclical process involving reflection, planning, learning, evaluation, and application [22]. Incorporating the CPD process of reflection and planning into the preceptor plan development assignments allowed participants to customize their preceptor development needs. Final preceptor development plans were submitted to the SSPPS faculty for assessment.

Participants were also coached on how to create structured rotation syllabi in alignment with the objectives for the CCHE/SSPPS post-graduate Pharm.D. IPPE and APPE rotations. Constructing rotation syllabi was included as a program outcome to achieve development of the participant’s envisioned Pharm.D. rotation by the end of the preceptor development program. Draft rotation syllabi were reviewed with feedback from peers and the SSPPS faculty before submission to the SSPPS faculty for final evaluation. Rotation syllabi were evaluated on alignment with IPPE or APPE specific rotation descriptions, objectives, schedule/calendar, prerequisites, and non-patient care assignments (e.g., monograph preparation, medication class review, journal club presentation).

On the final day of the program, participants completed case-based assessments from the perspective of a Pharm.D. preceptor. Participants were assessed on their ability to identify Pharm.D. learner strengths or weaknesses, apply CCHE/SSPPS Pharm.D. program specific assessment tools, provide feedback for the learner, or adjust the learning experience based on learner feedback as applicable to the learner case. Cases included components of professionalism and communication.

Participant performance on submitted preceptor development plans, rotation syllabi, and the case-based assessments contributed towards ultimate selection as a Pharm.D. preceptor at the CCHE. The SSPPS faculty evaluated each assessment using rubrics created for this preceptor development program. Table 2 summarizes the key themes within each assessment. A passing score of 70% was established to pass each assessment.

## 4. Results

Twenty-eight clinical pharmacists participated in the live training program. All participants held baccalaureate Pharmacy degrees. All participants submitted a final preceptor development plan and completed the learner case assessments on the final day. However, only twenty of twenty-eight participants (71%) submitted a completed rotation syllabus. Participants needed to earn a passing score of 70% in each assessment (Pharm.D. learner cases, individual preceptor development plan, and rotation syllabi) for selection to precept Pharm.D. students at the CCHE. Table 3 summarizes the participant pass rates of each evaluated area.

Most of the participants had never before created a rotation syllabus. Table 4 includes participants’ scores on individual components on their rotation syllabi [23]. Developing rotation site specific learning activities not directly related to patient care was identified as the most common area needing improvement.

Sixteen of the twenty-eight participants (57%) scored above 70% on all assessments. These 16 participants were recommended by the SSPPS faculty to precept Pharm.D. students at the CCHE. Six specialized in acute care, five specialized in ambulatory care, two specialized in compounding, one specialized in transitions of care, one specialized in pharmacogenomics, and one specialized in research. Participants that were not selected as Pharm.D. preceptors initially are encouraged to repeat Pharm. D. preceptor development training in the future.

After identification of the Pharm.D. preceptor group, specific APPE rotations for the CCHE/SSPPS Pharm.D. program were determined. APPE rotations in neuro-oncology, infectious diseases, bone marrow transplant, and the intensive care unit (ICU) were developed by the inpatient preceptors. Ambulatory care preceptors prepared Pharm.D. APPE rotations in the emergency room, multi-specialty clinic, general outpatient clinic, and day care unit. Specialty elective rotations were set up in the pharmacy managed pharmacokinetics laboratory, radiology pharmacy, nuclear pharmacy, and clinical nutrition.

Following the live training program, the selected CCHE Pharm.D. preceptors participated in ongoing virtual mentoring and preceptor development sessions with the three SSPPS faculty over the course of another 10 months. Each preceptor was assigned to present a learner case reflecting on their precepting skills including discussion on their own strengths and opportunities incorporating preceptor development concepts. The SSPPS faculty provided feedback on these preceptor-learner case presentations and peer discussion for modification of the learning experience/precepting approach was encouraged by the group.

The CCHE Pharm.D. preceptors also completed a survey to identify desired topics of interest for further preceptor development topics provided by the SSPPS faculty. Table 5 summarizes the identified topics. Navigating scenarios of challenging learners with the SSPPS faculty was desired by all surveyed preceptors.

## 5. Discussion

An analysis of the global pharmacy workforce published in 2016 found variations in availability of pharmacy workforce and provision of patient-centered care between countries and regions worldwide [24]. Helping to develop patient-centered pharmacy practices can help decrease the variation in pharmacy services around the globally. The University of Colorado SSPPS is committed to help develop the practice of pharmacy and Pharm.D. practitioners worldwide. Recently, the SSPPS created practice model development programs within ambulatory care provided in collaboration with the Qatar Primary Health Care Corporation [25].

The Pharm.D. preceptor development program described within this paper supports Pharm.D. training via preceptor development at the CCHE in alignment with the SSPPS mission of excellence and innovation in professional, graduate, and post-graduate education. Preceptor development programs are needed to ensure appropriate training of future pharmacy graduates to provide patient-centered pharmacy services globally. The FIP*Ed* Global Education Report 2013 found a shortage of clinical preceptors available internationally to educate pharmacy students in experiential rotations [3,26]. The FIP*Ed* 2016 Global Vision for Education and Workforce supports “all teachers and tutors have access to teacher training programmes and development in order to become high quality teachers and trainers for our profession [27]”.

Strengths of the CCHE/SSPPS preceptor development program included live delivery by experienced SSPPS clinical pharmacy faculty preceptors, active participation by the CCHE pharmacists to apply program content to their rotation plans and precepting styles, and opportunities for concurrent discussion with CCHE peers also completing the program. Live and virtual discussions were focused on familiarizing participants with experiential objectives within the Pharm.D. program and encouraging active interaction of students with other health care professionals during IPPE and APPE rotations. The CCHE pharmacists had been taught predominantly by observation models themselves so expecting active participation by Pharm.D. learners was a less frequently utilized precepting concept. While other described preceptor development programs included components of self-study for flexibility, ours was designed to maximize participant engagement with peers during the live program and virtual mentoring [28,29]. Participants informally expressed appreciation for the level of active participation in this program.

Preparing a rotation syllabus for their planned Pharm.D. rotations experiences contributed to the participants informally expressing feeling more prepared to take on the new Pharm.D. precepting expectations. Assessment of participants’ rotation syllabi also served as a peer review process and enabled SSPPS faculty to determine which participants understood the objectives of IPPE versus APPE rotations, set learner level appropriate expectations, planned time for routine feedback and evaluation of students, and expected the appropriate amount of non-patient care specific learning activities. These actions collectively are expected to enhance the quality of training and instill pedagogical rigor.

This program was successful in cultivating the development of Pharm.D. preceptors who worked in an exclusively pediatric population, focused on cancer. Nonetheless, this program was effective in training and identifying qualified and capable preceptors. Additionally, because the focus of this preceptor development was on training of Pharm.D. students’ skills, rather than knowledge, preceptor development was population and disease state agnostic.

Skills gained within the preceptor development program could be also applied beyond Pharm.D. student rotations. The CCHE also began offering pharmacy practice residencies in alignment with the American Society of Health-System Pharmacy (ASHP) Accreditation Standards for International Pharmacy Practice Residency Programs [30] in 2017. After the preceptor development training described within this paper, the majority of the CCHE Pharm.D. preceptors elected to precept both Pharm.D. and pharmacy practice residency learners at the CCHE. The SSPPS faculty observed the CCHE Pharm.D. preceptors frequently incorporating the preceptor roles (instruction, modeling, coaching, and facilitation) into their personal preceptor case presentations for the virtual mentoring assignments. Weitzel, et al., support the use of the preceptor roles for teaching clinical problem solving for both residency and student learners [17].

Limitations of this preceptor development program include the feasibility for other international pharmacy education programs to offer onsite preceptor development (due to cost, or enabling attendance by multiple participants during work hours) from experienced clinical pharmacy faculty preceptors, and challenges of providing ongoing training for the Pharm.D. preceptors. Group-based discussions were not as successful during the ongoing virtual sessions. The CCHE pharmacists were challenged to participate in the virtual training sessions after their work day and many CCHE preceptors had not yet precepted Pharm.D. students by the start of the virtual sessions limiting group discussions. A half-day onsite (or virtual) workshop at least six months after the initial program may be beneficial to facilitate preceptor attendance and generate group discussion. CCHE preceptors preferred actual case-based discussions of challenging scenarios the SSPPS preceptors had worked through over further didactic training during the ongoing virtual sessions. Additionally, providing ongoing mentoring from the SSPPS faculty on an as needed basis may be better suited than ongoing virtual group discussions.

There are several opportunities for improvement in further offerings of this preceptor development program. Shortening the length of the onsite program would decrease financial barriers. Developing and incorporating more video-based scenarios to help participants interpret and work through precepting scenarios would provide more opportunities for the application of skills included in the preceptor development program. Conducting a post training course evaluation and offering a half-day refresher and discussion workshop (onsite or virtually) may also be more feasible and beneficial for preceptors. The authors plan to revise and offer this preceptor development program for other interested international pharmacy programs.

Subsequent preceptor development is currently under development for the CCHE preceptors. A “train-the-trainer” approach utilizing the current Pharm.D. preceptors at the CCHE in collaboration with the SSPPS faculty to provide preceptor development for additional clinical pharmacists at the CCHE to become Pharm.D. preceptors is being considered. Finally, preceptors not initially selected to precept Pharm.D. students may benefit from peer mentoring from the current CCHE Pharm.D. preceptors, similar to the “preceptors-in-training” model in ASHP residencies [31].

## 6. Conclusions

This preceptor development program was successful in training and identifying Pharm.D. preceptors. These preceptors were familiarized with programmatic experiential expectations and facilitated the inclusion of students in the provision of advanced clinical pharmacy practice during experiential rotations. Development of individual preceptor development plans provided participants with opportunities for continuing professional development as they increase the precepting experience of Pharm.D. students. Evaluating experiential syllabi was needed for the selection of preceptors for this program to ensure that requirements and structure of the PharmD program was pedagogically sound.

## Figures and Tables

**Table 1:** University of Colorado Skaggs School of Pharmacy and Pharmaceutical Sciences (SSPPS) Preceptor Development Program for Potential Children’s Cancer Hospital of Egypt (CCHE) Pharm.D. Preceptors.

Day	Topic	Participant Assignments
1	**Introduction to Clinical Pharmacy** Definition of Clinical Pharmacy [13] JCPP Pharmacists’ Patient Care Process [12] ACCP Clinical Pharmacist Competencies [14] Entrustable Professional Activities [15] **Preceptor Development I** Effective preceptor characteristics [16] Preceptor roles [17] Didactic vs. experiential education IPPE/APPE objectives and expectations for CCHE/SSPPS Pharm.D.	Assignment #1: Prepare long and short-term precepting goals
2	**Preceptor Development II** Administrative skills Communication and feedback [18,19,20] Interprofessional team dynamics and communication with other healthcare providers. Preceptor continuing education opportunities, board certification.	Assignment #2: Create individual Preceptor Development Plan Reflect on personal communication style
3	**Practice Site Development and Rotation Design** Creating practice opportunities/Workflow incorporation of learners. Review types of clinical rotations Evaluation and assessment of learners Rotation design and syllabus development **Implementation** Orientation and training How to cultivate a successful preceptor	Assignment #3: Draft experiential syllabus
4	**Challenges of Precepting** Role delineation Conflict resolution [21] Challenging students/scenarios	Assignment #4: Revise and submit final preceptor development plan and rotation syllabus
5	**Final Evaluation:** case-based simulation with written evaluation	

**Table 2:** Assessment Themes.

Assessment	Key Assessment Themes
Learner Case Scenario #1	Participant identified multiple strengths and opportunities for improvement of the Pharm.D. learner Participant included feedback and specific strategies to enhance the Pharm.D. learner’s understanding and modify learner behavior
Learner Case Scenario #2	Participant identified areas within their rotation needing modification based on Pharm.D. learner feedback Participant identified which aspects of the rotation Pharm.D. learners valued Participant identified 2 specific modifications to make of their rotation Participant explained each planned modification
Preceptor Development Plan	Participant identified short- and long- term goals of their own development as a preceptor Participant provided 2 actionable and achievable items for each goal identified
Rotation Syllabus	Depending on type of rotation, the participant provided IPPE or APPE appropriate rotation: description objectives schedule/calendar pre-requisites non-patient care assignments

**Table 3:** Participant Pass Rates in Each Assessment.

Assessment	Number of Participants Completing Assessment	Participants Scoring ≥ 70%
Learner Case Scenario #1	28	86%
Learner Case Scenario #2	28	72%
Preceptor Development Plan	28	68%
Rotation Syllabus	20 ^1^	85%

^1^ Only twenty of twenty-eight participants (71%) submitted final rotation syllabi.

**Table 4:** Participants’ Scores on Rotation Syllabus Components.

Syllabus Components	Participants’ Mean Scores (n = 20)
Description	88%
Objectives	93%
Schedule/Calendar	86%
Prerequisites	86%
Non-patient Care Related Assignments	83%

**Table 5:** Preceptor Development Topic Interest by the CCHE Pharm.D. Preceptors.

Topic	Preceptors Interested in Topic (n = 16)
Incorporating Journal Club Activities into Rotation	88%
Assessing Baseline Knowledge of Trainee	94%
Preceptor Role: Instruction	50%
Preceptor Role: Modeling	63%
Preceptor Role: Coaching	63%
Preceptor Role: Facilitating	75%
Navigating Problematic Learner Cases	100%
